# An Iowa Girl’s Ambition

**Published:** 1882-01

**Authors:** 


### AN IOWA GIRL'S AMBITION.

SMARTEST girl I've met in Iowa I met yesterday at Nevada, Story County, Northwestern Iowa — Miss Belle Clinton. Miss Clinton is a bright-eyed, rosy-cheeked girl of about twenty, as full of fun and health and vigor as a good girl can be. Two years ago Miss Clinton was a school teacher. Saving up by her teaching about $160, she last spring borrowed a span of horses from her father, rigged up a "prairie schooner," and, taking her little brother, started for Dakota. Miss Clinton says laughingly to-day, speaking of her trip: "Why, I never lived so nicely in my life, and I never had such an appetite, and such courtesy I received everywhere! Rough, rude men would come up to our camp, and, after I had talked to them awile, offer to build my fire and actually bring water to me. We went up through the wheat country, which they call the 'Jim River country.' It's about 100 miles east from the Missouri at Fort Sully. I homesteaded 160 acres of land. Then I took up a timber claim of 120 acres more."

"What is a timber claim?"

"Why, I hired a man and we set out ten acres of trees. This gave me 160 acres more; so I have 320 acres now. But I must tell you about those trees. They were young locust, apple and black walnut sprouts. I sowed a peck of locust beans, a pint of apple seed and two bushels of black walnuts in our garden in Iowa a year ago. These sprouts were little fellows and we could set them out fast—just go along and stick them in the ground. But they are just as good. I believe my 3,000 little black walnut sprouts will be worth $15 apiece in ten years, and $20 apiece in fifteen. My locust trees will some time fence the whole country."

"Then what did you do?"

"We built a shanty and broke up five acres of land; and this fall we came back to Iowa to spend the winter, and here we are. In the spring I'll go back with more black walnut and locust sprouts and take up 160 acres more. The trees are just what I want to plant anyway, and they'll pay better than any wheat crop that could be raised—only I've got to wait for them ten or twelve years; but I can wait."

Here is a girl who owns in her own right 320 acres of splendid black prairie soil now and who will own 480 acres in the spring, every acre of which will bring $5 within three years and $10 within five years and $20 within ten years. Her black walnut and locust trees will be worth as much more. At thirty she will be worth $25,000.—*Letter to Chicago Tribune.*

